# Susceptibility genes of hyperuricemia and gout

**DOI:** 10.1186/s41065-022-00243-y

**Published:** 2022-08-04

**Authors:** Yue-Li Nian, Chong-Ge You

**Affiliations:** Laboratory Medicine Center, Lanzhou University Second Hospital, Lanzhou, 730030 China

**Keywords:** Hyperuricemia, Gout, Susceptibility gene, Single nucleotide polymorphism, Serum uric acid

## Abstract

Gout is a chronic metabolic disease that seriously affects human health. It is also a major challenge facing the world, which has brought a heavy burden to patients and society. Hyperuricemia (HUA) is the most important risk factor for gout. In recent years, with the improvement of living standards and the change of dietary habits, the incidence of gout in the world has increased dramatically, and gradually tends to be younger. An increasing number of studies have shown that gene mutations may play an important role in the development of HUA and gout. Therefore, we reviewed the existing literature and summarized the susceptibility genes and research status of HUA and gout, in order to provide reference for the early diagnosis, individualized treatment and the development of new targeted drugs of HUA and gout.

## Introduction

**Fig. 1:**
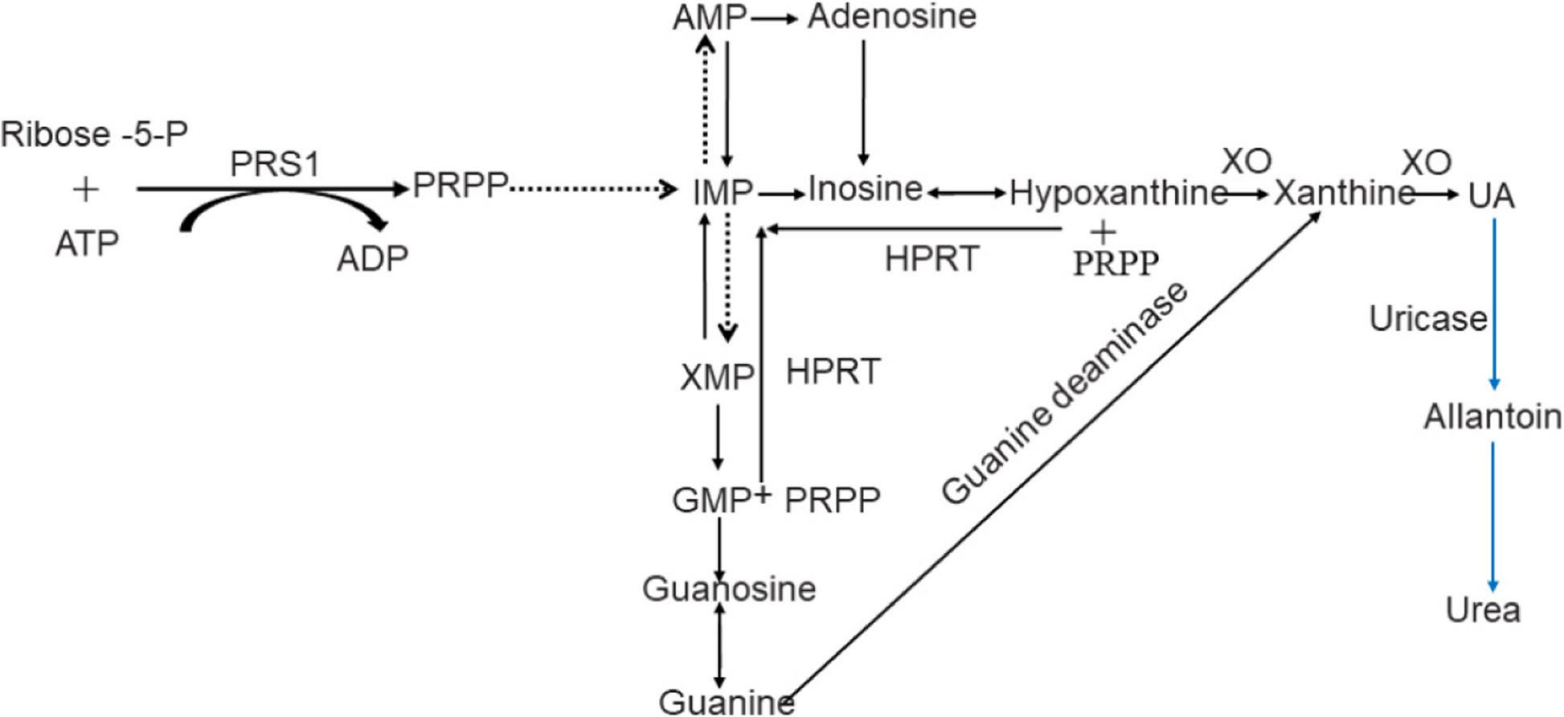
Uric acid metabolism diagram. The solid line represents one-step reaction and the dotted line represents multi-step reaction, the blue part is mainly found in most mammals except humans

Gout is a common disease caused by purine metabolism disorder, which is primarily caused by the accumulation of uric acid (UA) crystals in joints and other tissues. It is typically characterized by recurrent episodes of acute inflammatory arthritis, and the metatarsophalangeal joint of the big toe is the most vulnerable part [1]. The occurrence of gout is often significantly correlated with the increase of serum uric acid (SUA) levels. In most mammals, UA is oxidized by uricase to a more water-soluble allantoin, which is excreted from the kidney (Fig. 1). However, in the process of human evolution, due to the silent mutation of the gene encoding uricase, UA becomes the final product of purine metabolism in humans, and its concentration is 3 to 10 times that of other mammals [2]. When the concentration of SUA in human exceeds 420 μmol/L (male) or 360 μmol/L(female) was defined as HUA. HUA plays a crucial role in the occurrence and development of gout. It has been reported that about a quarter of patients with HUA will develop gout [3]. Chronic gout can lead to lifelong disability. Moreover, studies have shown that the heritability of SUA is about 73% [4], which suggests that HUA and gout are largely determined by genetic factors. Therefore, it is significant to explore HUA and gout from the perspective of genetic variation.

**Fig. 2:**
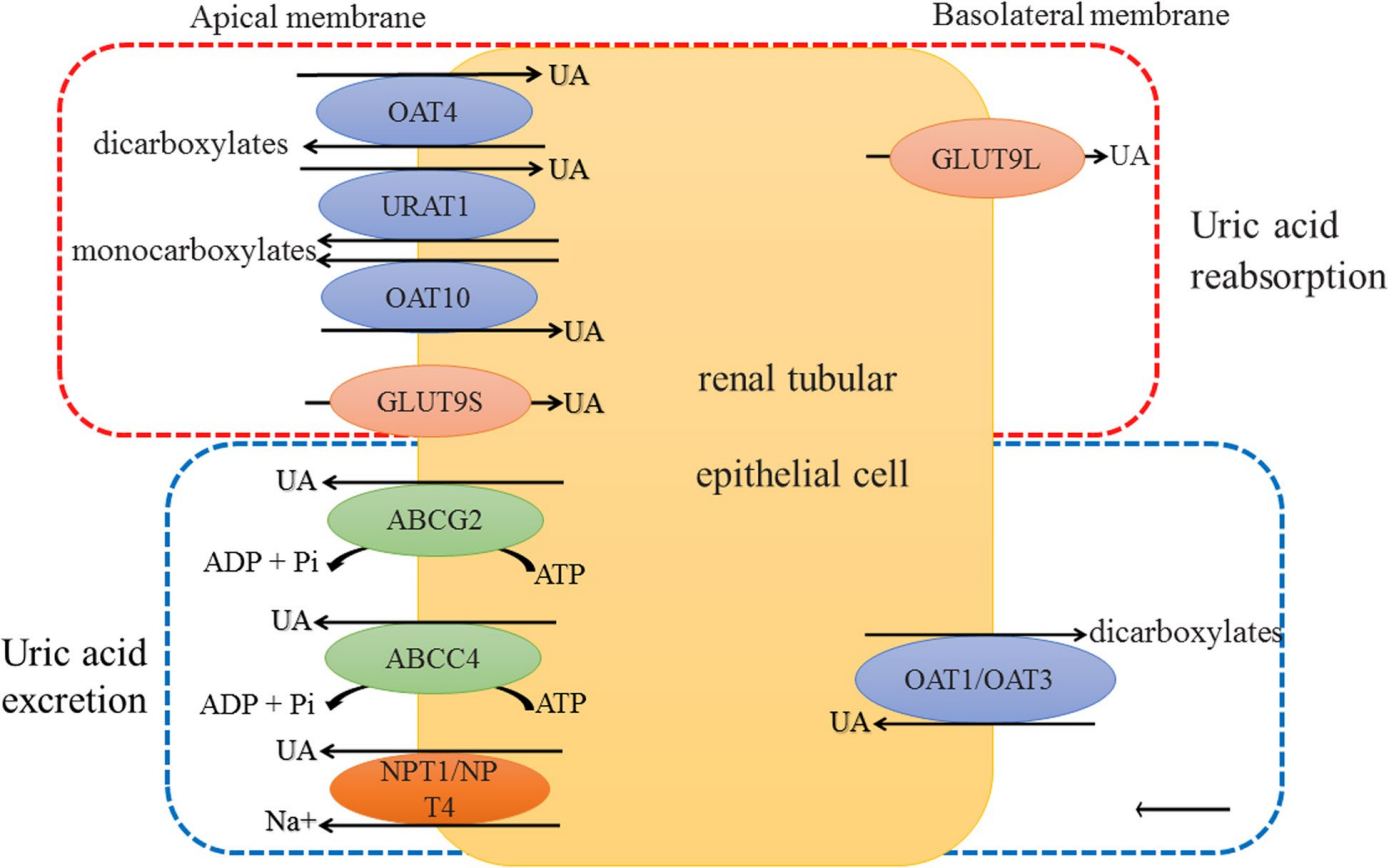
UA transport proteins on the membrane of renal tubular epithelial cells. In the red square frame are transport proteins involved in UA reabsorption. In the blue square frame are transport proteins involved in UA excretion

UA is mainly produced by the liver, two-thirds of which is excreted via the kidney and one-third via the intestine [5]. Among them, HPRT and PRS1 are the most important enzymes involved in liver UA production (Fig. 1); while GLUT9, ABCG2 and OATs, etc. are the main transporters involved in the reabsorption and excretion of UA in the kidney and intestine (Fig. 2). Studies have shown that *HPRT* and *PRPS1* gene mutations seem to be the main cause of primary gout [6]; *SLC22A11* gene mutation is associated with RUE (renal underexcretion) gout [7]; ABCG2 seems to be one of the reasons for the genetic heterogeneity of ROL (renal overload) and RUE gout [8]. It can be seen that any abnormality of enzymes or transporters involved in UA metabolism and their upstream genes will affect SUA levels. Consequently, this paper reviews the genes involved in HUA and gout mainly from three aspects (Table 1): UA production, UA reabsorption and UA excretion.

**Table 1:** Susceptibility Genes of HUA and Gout

Classification	Gene name	Gene product	Location (human)	Tissue distribution	Refs
Production	*HPRT1*	HPRT1	Xq26.2-q26.3	Multi-tissue expression	NCBI, [9]
	*PRPS1*	PRS1	Xq22.3	Multi-tissue expression	NCBI, [10]
	*ALDH16A1*	ALDH16A1	19q13.33	High expression in kidney	NCBI, [11]
Reabsorption	*SLC22A11*	OAT4	11q13.1	Kidney	NCBI, [12, 13]
	*SLC22A12*	URAT1	11q13.1	Kidney	NCBI, [13]
	*SLC22A13*	OAT10	3p22.2	Kidney	NCBI, [14]
	*SLC2A9*	GLUT9	4p16.1	Liver and kidney	NCBI, [15]
Excretion	*ABCG2*	BCRP	4q22.1	Kidney and other tissues	NCBI, [16]
	*ABCC4*	MRP4	13q32.1	Kidney and other tissues	NCBI, [17]
	*SLC22A6*	OAT1	11q12.3	Kidney and other tissues	NCBI, [13, 18]
	*SLC22A8*	OAT3	11q12.3	Kidney and other tissues	NCBI, [13, 18]
	*SLC17A1*	NPT1	6p22.2	Kidney and other tissues	NCBI, [12]
	*SLC17A3*	NPT4	6p22.2	Kidney and other tissues	NCBI, [12, 19]
	*SLC17A4*	NPT5	6p22.2	Kidney and other tissues	NCBI, [12, 20]
	*SLC2A12*	GLUT12	6q23.2	Kidney and other tissues	NCBI, [21]
Other	*PDZK1*	Various scaffold proteins	1q21.1	Liver, kidney and other tissues	NCBI, [22]
	*GCKR*	GKRP	2p23.3	Liver	NCBI
	*PKD2*	Polycytin-2	4q22.1	Multi-tissue expression	NCBI, [23, 24]
	*SLC16A9*	MCT9	10q21.2	Kidneys and other tissues	NCBI, [12]
	*CARMIL1*	CARMIL1	6p22.2	Kidney and other epithelial tissues	NCBI, [25]
	*SCGN*	Secretagogin	6p22.2	Neuroendocrine tissue and pancreas	NCBI, [12]
	*UMOD*	THP	16p12.3	The major secretory protein in urine	NCBI, [26, 27]

### Genes related to UA production

#### *HPRT*

*HPRT* gene is located on human chromosome X (xq26.2-q26.3), with a total length of 44 kb, including 9 exons and 8 introns, encoding hypoxanthine guanine phosphoribosyltransferase (HPRT) [9]. As shown in Fig. 1, HPRT is the most important enzyme in the purine salvage pathway, which catalyzes the synthesis of hypoxanthine into hypoxanthine nucleotides and the conversion of guanine into guanine nucleotides. Its activity is regulated by the synergistic effect of guanine and IMP [28]. *HPRT* gene mutation can cause HPRT enzyme activity defect, then it will lead to the surplus of its substrates hypoxanthine and guanine, and these surplus purines will be converted into UA under the action of xanthine oxidase (XO) (Fig. 1), resulting in the increase of UA levels in the body [29], and finally cause gout. Clinically, the disease caused by *HPRT* deficiency belongs to X-linked genetic disease, which mainly affects men [30], and the severity of the disease is positively correlated with the degree of enzyme deficiency [31]. Moreover, diseases caused by *HPRT* gene mutations can be divided into three types according to the degree of enzyme deficiency: the most serious one is Lesch-Nyhan syndrome (LND) with enzyme activity less than 1.5%, mainly manifested in HUA, abnormal development of nervous system, involuntary movement, and self-injurious behavior; however, 1.5–2% of patients with enzyme activity showed HUA with neurological dysfunction; in addition, Keeley-seegmiller syndrome with enzyme activity of 8%—60% only shows HUA related symptoms [31]. Recently, studies have found that *HPRT* pathogenic mutants c.103G > A (p.V35M) [12], c.277-281delATTGC, c.299 (exon 3) T > A, c.468-470delGAT and loss (exon: 6) 84 bp are related to family juvenile gout. [6]. The interaction between *HPRT* gene mutants and β-amyloid precursor protein (*APP*) gene regulate the epigenetics of LND by affecting alternative APP pre-mRNA splicing [32]. The increase of SUA caused by *HPRT* deficiency is regulated by GLUT9 single nucleotide polymorphism (SNP) [5]. *P53* up-regulates the expression of *HPRT* [33]; miR-181a down-regulates the expression of *HPRT* [34]. It can be seen that *HPRT* pathogenic mutants are significantly associated with familial juvenile gout. Therefore, it is particularly important to detect *HPRT* gene in these patients.

#### *PRPS1*

*PRPS1* gene is located on human chromosome X (Xq22.3), encoding phosphoribosyl pyrophosphate synthase 1 (PRS1), which is involved in human nucleotide synthesis via catalyzing the synthesis of phosphoribosyl pyrophosphate (PRPP) by adenosine triphosphate (ATP) and 5-phosphoribosyl (R-5P) (Fig. 1) [10]. *PRPS1* is transcriptionally regulated by miR-p376 [35], whose accelerated transcription will lead to the superactivity of PRS1 and eventually cause the increase of UA synthesis [36]. In general, pathogenic mutants of *PRPS1* cause hereditary gout, Arts syndrome, Charcot-Marie-Tooth neuropathy type 5 (CMTX5) and X-linked deafness 1 (DFNX1), and mainly affect men [37]. Recently, Zikanova et al. [38] found a new mutation of *PRPS1*: c.520 G > A (p.G174R) leads to PRS1 hyperactivity, then resulting in severe HUA. In addition, Yang et al. [36] also found another missense mutation of *PRPS1*: c.521(exon)G > T, p. (Gly174Val) is associated with HUA and gout. However, studies have found that *PRPS1* missense mutant c.359G > T (p.Gly120Val) causes a rare adult-onset cerebellar ataxia in female [37], and *PRPS1* mutant c.82 G > C causes optic atrophy and deafness [39]. It can be seen that only the mutations that causes the superactivity of *PRPS1* will increase the synthesis of UA. Therefore, the possibility of *PRPS1* gene mutation cannot be ruled out when SUA levels is normal. Furthermore, the detection of *PRPS1* activity is great significance for the early diagnosis of HUA and gout. *PRPS1* may be a potential target for the treatment of HUA and gout in the future. Because this gene mutation is more likely to occur in early-onset gout, thus, young patients with simple HUA should be screened for *PRPS1* mutation.

#### *ALDH16A1*

*ALDH16A1* gene is located on human chromosome 19q13.33 and consists of 17 exons, encoding acetaldehyde dehydrogenase 16 family A1 (ALDH16A1) [11]. It is highly expressed in kidney [40] and catalyzes a variety of aldehyde reactions [11]. Leask et al. found that *ALDH16A1* rs150414818 (Pro476Arg) mutation disrupted the interaction between *ALDH16A1* and *HPRT*, thereby affecting purine metabolism, resulting in elevated UA [41]. In mice, knockdown of *ALDH16A1* resulted in decreased *SLC17A3* expression and increased *SLC16A9* and *ABCC4* expression [41]. Therefore, *ALDH16A1* may be involved in the regulation of SUA via interacting with other UA transporters.

### Genes related to UA reabsorption

#### Solute carrier family 22 (SLC22A)

##### SLC22A11

*SLC22A11* gene is located on chromosome 11q13.1, encoding organic anion transporter 4 (OAT4) and is expressed in the apical membrane of renal proximal tubular epithelial cells. OAT4 is an asymmetric UA transporter with 53% homology with URAT1 [12]. It reabsorbs UA in the form of exchange between organic anions and dicarboxylate (Fig. 2) [12, 13]. The expression of OAT4 is regulated by *PDZK1*, *NHERF1* and protein kinase C [13, 42]. IL-23 down-regulates OAT4 mRNA expression [43]. In addition, the inhibition of Wnt signaling pathway down-regulates the expression of OAT1, OAT3 and OAT4 [42]. GWAS have revealed that *SLC22A11* rs17300741 was associated with SUA levels, while rs2078267, rs2186571, rs17299124 and rs17300741 were associated with gout [44]. Among them, rs17300741 is dramatically associated with RUE gout in Japanese population [7], but whether this association exists in other regions has not been confirmed.

##### SLC22A12

*SLC22A12* gene is located on chromosome 11q13.1 and encodes urate transporter 1 (URAT1), which is expressed in the apical membrane of renal tubular epithelial cells. URAT1 is a high affinity UA transporter, which absorbs UA from raw urine and plays an important role in maintaining human UA homeostasis. Like OAT4, URAT1 is also an asymmetric UA transporter [13], which participates in the reabsorption of UA through monocarboxylate exchange (Fig. 2). *SLC22A12* gene dysfunctional mutations cause URAT1 dysfunction, then leading to hereditary renal hypouricemia type 1 (RHUC1), which is characterized by decreased SUA levels and increased UA excretion [45]. Epidemiological investigation showed that 90% of hypouricemia (SUA ≤ 2.0 mg/dl) was caused by nonfunctional URAT1 mutations [46]. The rare variant of *SLC22A12* gene is considered to have strong ethnic specificity [47]. *SLC22A12* rs559946 is associated with a higher risk of gout in the Han population; rs3825017 is associated with gout risk in Czech population; rs75786299, rs7929627 and rs3825017 are associated with HUA in Korean population [37]; rs11231825 (p.H142H) is related to gout susceptibility in Vietnamese population [48]. *SLC22A12* rs121907896(p.R90H) and rs121907892 (p.W258X) are the two most common variants leading to hypouricemia in the Japanese population [49, 50]. Sakiyama et al. [50] proved that these two variants were protective factor for HUA and gout. Consistent with previous studies, pavelcova et al. also found that *SLC22A12* gene variant rs3825017 (p.N82N) increased the risk of gout [51]. However, Toyoda et al. [53] found that dysfunctional mutations of *SLC22A12* gene have prominent anti-gout effect. Even in the presence of *ABCG2* pathogenic mutations, these mutations still have a protective effect on gout. In addition, they found that the protective effect of *SLC22A12* on gout exceeded the pathogenic effect of *ABCG2* on gout. Meta-analysis showed that *SLC22A12* rs3825016 and rs3825018 are risk factors for gout and HUA, while rs475688 is a protective factor for HUA [52]. It can be seen that the vast majority of *SLC22A12* gene mutations inhibit the function of URAT1 and reduce the risk of gout. In addition, 27-Hydroxycholesterol (a metabolite of cholesterol) can activate *SLC22A12* gene promoter via estrogen response elements (EREs), and then up-regulate the expression of *SLC22A12 *[53].

##### SLC22A13

*SLC22A13* gene is located on chromosome 3p21.3, which encodes organic anion transporter 10 (OAT10). It is expressed in the apical membrane of proximal tubular epithelial cells [14]. In vitro analysis showed that OAT10 is a low affinity UA transporter, which has 44% homology with OAT1 and is mainly involved in the reabsorption of UA (Fig. 2) [54]. Insulin can selectively activate its UA transport function [55]. Bahn et al. [54] found that the expression of *SLC22A13* in chickens was gender dependent, and the female was higher than the male. However, this gender dependent expression does not seem to exist in humans, because the SUA levels of men is higher than women. Recent studies have also shown that dysfunctional missense mutation of *SLC22A13* gene reduced SUA levels and the risk of gout. Meta-analysis displayed that rs117371763 (R377C) variant of *SLC22A13* gene has significant anti-gout effect [56]. It is certain that *SLC22A13*, like *SLC22A12*, can provide effective targets for the treatment of gout.

#### SLC2A9

*SLC2A9* gene is located on chromosome 4p16.1 and has 13 exons, encoding glucose transporter 9 (GLUT9) with strong UA transport capacity, which is mainly expressed in liver and kidney [15]. Human GLUT9 has two subtypes: GLUT9L and GLUT9S. In proximal tubular epithelial cells, GLUT9L expressed in the basolateral membrane is the only UA efflux transporter [57](Fig. 2); GLUT9S expressed in the apical membrane regulates the reabsorption of UA together with URAT1 [58] (Fig. 2). Therefore, the loss of GLUT9 function will completely inhibit the outflow of UA, thus blocking the reabsorption of UA by the apical membrane UA transporter. It is well known that *SLC2A9* gene mutation causes hereditary renal hypouricemia type 2 (RHUC2), which is characterized by severe hypouricemia and easy to be complicated with acute renal failure and renal calculi. Windpesl M et al. found that *SLC2A9* gene mutation is a cause of RHUC2 in Austrian native families, especially homozygotes will have severe hypouricemia, and carriers have a higher risk of acute renal injury (AKI) [59]. Moreover, the CC genotype of *SLC2A9* SNP rs1172228 in gout patients is significantly associated with renal calculi in Malaysian population [60]. However, consistent with previous results, two variants of *SLC2A9* gene (p.V282I:rs16890979 and c.1002 + 78A > G:rs6823877) may be protective factors of gout [51]. Moreover, *SLC2A9* SNP rs62293298 attenuates the risk of HUA [61]. In addition, *SLC2A9* SNPs affect gout caused by HPRT deficiency and the therapeutic response of allopurinol [5]. Meta-analysis showed that SNP rs16890979, rs1014290and rs12510549 of *SLC2A9* could prevent gout. Among them, rs16890979 was associated with lower gout risk in Caucasians and Asians, rs1014290 was associated with lower gout risk in Asians, and rs12510549 was associated with lower gout risk in Caucasians [62]. *SLC2A9* rs3733591 (Arg265His) variant increases the risk of gout [45]. *SLC2A9* rs 737267, rs6449213 and rs1014290 are associated with gout in the UK, German and Croatian populations, respectively [45]. *SLC2A9* rs3775948G and rs13129697G alleles reduce the risk of HUA [63]. Therefore, *SLC2A9* SNPs may have a protective effect on gout, but its severe hypouricemia and its complications may endanger the lives of patients. Non-additive genetic interaction between *SLC2A9* and insulin related genes also affects SUA [55]. Moreover, this effect is most obvious in women, which is consistent with the greater effect of *SLC2A9* on UA in women. Insulin promotes the activity of various UA transporters via activating MAPK p38, MAPK p44/42 and Akt pathways [55]. E4 promoter- binding protein 4 (*E4BP4*) gene directly binds P2 promoter to down-regulate the expression of *SLC2A9* in mouse liver [64].

### Genes related to UA excretion

#### *ABCG2*

ATP-binding cassette (ABC) transporters belong to the transmembrane protein family and are divided into seven subfamilies: A-G. At present, it is known that there are five members of ABCG subfamily: *ABCG1*, *ABCG2*, *ABCG4*, *ABCG5* and *ABCG8 *[16]. Among them, the *ABCG2* gene is located in chromosome 4q22.1, which consists of 16 exons and 15 introns, encoding ABC transporter G2(ABCG2), also known as breast cancer resistance protein (BCRP). ABCG2 is an ATP dependent exogenous transporter, which mediates the excretion of UA (Fig. 2) [65, 66]. Therefore, *ABCG2* dysfunction will increase the risk of HUA and gout. Progesterone response factor down-regulates the expression of ABCG2, while estrogen response element up-regulates its expression [67]. GWAS showed that the genetic variation of *ABCG2* seems to be one of the reasons for the genetic heterogeneity of ROL and RUE gout [8]. Its pathogenic mutants are considered to be the strongest genetic risk factor for RUE gout and HUA [68]. Among them, rs2231142 (Q141K) variant reduces its allele expression in the kidney and block the excretion of intestinal UA [41]. Furthermore, rs2231142 has gene dose effect on gout [61]. In Xenopus oocytes, insulin could up-regulate the transport activity of ABCG2, but does not affect the transport activity of Q141K variant [55]. In the mouse model, knock-in *ABCG2* Q141K variant could down-regulate the expression of ABCG2 in male mice without affecting female mice [69]. *ABCG2* rs372192400 (R147W), rs753759474 (T153M), rs752626614 (F373C) and rs200894058 (S572R) could down-regulate the expression of ABCG2 [68]. *ABCG2* rs2054576 is related to HUA in the Korean population [70]; rs72552713 is associated with gout susceptibility in Vietnamese population [48]; c.725 T > C (p. I242T) is involved in the occurrence of early-onset HUA and gout [71]. Moreover, the more pathogenic variants carrying *ABCG2*, the earlier the onset of HUA and gout [68]. Interestingly, meta-analysis showed that *ABCG2* SNP rs2231137 (p.V12M) was a protective factor for gout [72]. The genotype combination of mutants Q141K and Q126X can be used to evaluate ABCG2 activity [73]. The association of A1CF variation and BAZ1B variation with HUA and gout has also been concerned recently. Intriguingly, these two new variants appear to be associated with *ABCG2* dysfunctional variants. In other words, when *ABCG2* dysfunctional variation and A1CF variation exist at the same time, A1CF variation is significantly correlated with gout, but in the absence of *ABCG2* variation, the correlation between A1CF variation and gout is no longer significant. However, the BAZ1B variation has a significant correlation with gout with or without *ABCG2* dysfunctional variation [74]. It can be seen that *ABCG2* gene variants and their SNPs are not only risk factors for HUA and gout, but also increase the risk of HUA and gout via interacting with other gene variants.

#### *ABCC4*

*ABCC* is the largest subfamily of ABC Family with 9 members. *ABCC4* gene is located on chromosome13q32.1 [17]and encodes multidrug resistance protein 4 (MRP4) [75]. It is mainly expressed in the basolateral membrane of hepatocytes and apical membrane of proximal renal tubular epithelial cells [76]. MRP4 is an ATP dependent unidirectional efflux pump, which can participate in the excretion of UA in proximal tubules in coordination with BCRP [77] (Fig. 2). miR-124a and miR-506 down-regulate the expression of *ABCC4 *[78]. In poultry, knockdown of *ABCC4* in proximal tubules reduced UA secretion by 80% [77]. It can be seen that ABCC4 is the key transporter of UA excretion in poultry kidney. Afterwards, Tanner et al. [79] repeated sequencing of *ABCC4* in patients with HUA in New Zealand Māori and Pacific, identified a common variant SNP rs4148500 and a rare variant P1036L that were significantly associated with HUA and gout. They also found that the transport activity of MRP4 seemed to be affected by elevated UA levels, because the UA transport activity of MRP4 in individuals with P1036L mutation decreased by 30% compared with normal controls. Obviously, ABCC4 plays a key role in maintaining UA homeostasis.

#### *SLC22A6* and *SLC22A8*

Human *SLC22A6* and *SLC22A8* genes are located on chromosome 11q12.3. The former encodes organic anion transporter 1 (OAT1) and the latter encodes organic anion transporter 3 (OAT3). In the kidney, immunohistochemistry showed that both OAT1 and OAT3 were located in the basolateral membrane of proximal tubular epithelial cells [13, 18]. OAT1 and OAT3 not only show overlapping substrate specificity, but also share transportation mode and driving force. They are famous multi-specific drug transporters [80]. The expression of OAT1 and OAT3 are regulated by protein kinase A and C [42]. Inhibition of Wnt signaling pathway down-regulates the expression of OAT1 and OAT3 [42]. Hepatocyte nuclear factor 1-α significantly up-regulates the expression of OAT1 in mouse kidney [81]. Estrogen receptor-α (ER-α) indirectly induces the transcriptional expression of OAT1 [82]. cAMP-response element(CRE) regulates the constitutive expression of human *SLC22A8* gene [83]. Previous studies have shown that UA is the endogenous substrate of OATs [18]. OAT1 and OAT3 participate in the excretion of SUA through UA/dicarboxylate exchanger [84] (Fig. 2). Existing studies have shown that the expression of OAT1 and OAT3 is decreased in HUA. Recently, it was found that alcohol-soluble extract increases the expression of OAT1 and reduces the expression of URAT1, so it has significant anti-gout effect and does not affect renal function [85]. In addition, the study found that total flavonoids of S. glabra has a significant UA lowering effect in mice, because it can not only up-regulate the expression of OAT1 in kidney, but also inhibit xanthine oxidase [86]. Although *SLC22A6* and *SLC22A8* play a key role in UA transport, the specific mechanism of these two genes in HUA and gout still needs to be further studied, so as to provide new targets for the treatment of HUA and gout.

#### *SLC17A*

*SLC17A* family transporters are Na^+^ dependent phosphate transporters, which can mediate the transmembrane transport of organic anions and coordinate UA excretion [87]. Up to now, there are three major genes in *SLC17A* family involved in UA transport (Fig. 2): *SLC17A1*, *SLC17A3* and *SLC17A4*, which all located on chromosome 6p22.2. Among them, sodium dependent phosphate transporter 1 (NPT1), encoded by *SLC17A1*, is located in the apical membrane of renal proximal tubular epithelial cells [12]. E4BP4 down-regulates the expression of *SLC17A1* in mouse liver [64]. Sodium dependent phosphate transporter 4 (NPT4), encoded by *SLC17A3*, is mainly expressed in the liver and kidney and is involved in the secretion of UA (Fig. 2) and the elimination of various anionic drugs [19]. *SLC17A4* encodes sodium dependent phosphate transporter 5 (NPT5), which is mainly expressed in pancreas, liver and intestine [20], but weakly expressed in kidney [12]. Recently, it was found that *SLC17A1* and *SLC17A3* SNPs are related to SUA levels, which may be involved in the occurrence of gout [65]. *SLC17A1* rs1165196 significantly enhances UA secretion and reduces the risk of RUE gout; while rs9393672 and rs942379 are significantly correlated with female SUA [44].

#### *SLC2A12*

*SLC2A12* encodes glucose transporter 12 (GLUT12), which belongs to the same family as GLUT9. It is a physiological UA transporter and is widely expressed in liver and kidney [21]. GLUT12 is a sodium independent bidirectional UA transporter, which may be involved in the transport of UA from blood to liver. In the mouse model of HUA, knockout of *SLC2A12* gene causes *SLC2A12* dysfunction, which leads to the increase of SUA levels [21]. It can be seen that *SLC2A12* deletion mutations may increase the incidence of HUA and gout.

### Other genes involved in UA regulation

#### *PDZK1*

PDZ domain-containing 1 (PDZK1) is a scaffold protein located on chromosome 1q21.1 that regulates SUA levels via participating in the assembly of renal UA transporter complex [22]. Although PDZK1 is not directly involved in UA transport, it interacts with C-terminal of various UA transporters, thereby regulating the expression of related proteins [41]. In human embryonic kidney 293 cells (HEK293 cells), co-expression of PDZK1 and URAT1 enhances the transport capacity of UA [77]. *PDZK1* rs12129861 is considered as a risk allele for gout [88]. *PDZK1* rs1967017 up-regulates the expression of *PDZK1* via altering the transcription factor binding site of HNF4A [89]. Long noncoding RNA (lncRNA) PENG up-regulates the expression of *PDZK1* via secreting miR-15b [90]. In addition, *ABCG2* and *PDZK1* gene-gender interactions are associated with gout risk in European populations [91].

#### *PKD2*

Like *ABCG2*, *PKD2* gene is also located on chromosome 4q22.1 [23, 24], encoding ion channels of transient receptor potential superfamily (*TRPP2*, *PKD2*, *PC2* or polycystin-2) [92]. It is related to the development, morphology and function of renal tubules and participates in the regulation of intracellular calcium homeostasis and other signal transduction pathways [93].Studies have confirmed that the epistatic interaction between *PKD2* and *ABCG2* is associated with the risk of HUA and gout [94]. The interaction between *PKD2* SNP rs2725220 and nutritional factors increases the risk of HUA and gout in Koreans [95]. In addition, *PKD2* expressed in B cells may be involved in B cell-mediated gout inflammation [96].

#### *SLC16A9*

*SLC16A9* gene encodes monocarboxylic acid transporter 9 (MCT9), which is mainly expressed in kidney, parathyroid gland, trachea, spleen and adrenal gland [12]. MCT9 is mainly involved in the reabsorption of renal UA [41], and its activity is regulated by extracellular H^+^ and Na^+^ [97]. In addition, *SLC16A9* SNPs are closely related to the occurrence and development of gout. Among them, rs2242206 reduces UA excretion in the intestine, which is significantly correlated with ROL gout, while rs550527563 is dramatically correlated with early-onset gout [98, 99]. Although it has been confirmed that there is a remarkable correlation between *SLC16A9* gene and different types of gout, its specific regulatory mechanism is not clear. This suggests that if we can clearly clarify the specific mechanism of *SLC16A9* on gout in future research, which may provide an effective target for the precise treatment of gout.

#### *CARMIL*(*LRRC16A*)

*CARMIL* gene is located on chromosome 6p22.2 and encodes myosin I connexin (CARMIL). It is expressed in kidney and other epithelial tissues and participates in the maintenance of cell shape [25]. *CARMIL* affect the activity of actin, which interacts with UA transporter and scaffold protein on renal apical membrane, so as to affect the function of UA transporter and indirectly cause the change of SUA levels [100]. A meta-analysis showed that *LRRC16A* was related to UA concentration [12]. Subsequently, Sakiyama et al. [100] found that *LRRC16A* SNP rs742132 was related to gout susceptibility in Japanese population. Sakiyama and others researchers believe that *LRRC16A* may participate in the occurrence and development of gout by affecting the function of UA transporter. However, there are few studies on the relationship between this gene and gout, and the specific regulatory mechanism is not clear.

#### *SCGN*

SCGN gene is located on chromosome 6p22.2. It encodes secretagogin,which is mainly expressed in neuroendocrine tissues and pancreatic β cells [12]. GWAS showed that *SCGN* was correlated with SUA levels [12]. In addition, studies on the change of SUA levels caused by this gene mutation have been reported [101]. However, the relationship between *SCGN* gene and gout has not been reported.

#### *MAF*

*MAF* is a transcription factor [102] involved in the regulation of SUA, which is highly expressed in human and mouse kidneys [103]. *MAF* gene expression is regulated by two independent upstream genetic signals, of which lncRNA is the most prominent [41]. It not only affects the structure and function of kidney, but also participates in the regulation of renal urate, and is related to SUA and gout susceptibility [104]. Recently, Higashino et al. [104] found that a common variant rs889472 of *c-MAF* was related to gout susceptibility in Japanese men through univariate logistic regression analysis.

#### *UMOD*

Uromodulin (UMOD) is encoded by *UMOD* gene located on chromosome 16p12.3, also known as Tamm-Horsfall protein (THP). It is the major protein secreted in normal urine [26, 27]. Its expression is regulated by transcription factors such as SP1, TP3, POU2F1, STAT3 and RARA [105]. Researchers found that more than 90% of *UMOD* gene mutations occurred in exons 3 and 4 [27]. This mutation causes autosomal dominant tubulointerstitial kidney disease (ADTKD-UMOD), also known as familial juvenile HUA nephropathy (FJHN) [27, 106]. This disease is an autosomal dominant disease, which is rare in children. It is mainly characterized by HUA, gout and chronic progressive nephropathy [107]. Interestingly, recently, ADTKD-UMOD caused by a new mutation of *UMOD* gene (c.1648G > A, p.V550I) was found in a 3-year-old Chinese boy [108], and the child showed persistent hematuria. On the contrary, a new *UMOD* gene mutation (c.163 g > A) was recently identified in the Brazilian family. Although it is related to ADTKD, the affected members do not seem to show HUA and gout [109]. In addition, homozygous mutations in *UMOD* gene seem to be more prone to early-onset gout [27]. The study found that the methylation level of UMOD in peripheral blood was related to the risk of gout, and its methylation evaluation could predict the risk of gout [110].

#### *ALDH2*

Aldehyde dehydrogenase 2 family member (*ALDH2*) gene is located on chromosome 12q24.12 and encodes aldehyde dehydrogenase 2 (ALDH2), which participates in alcohol metabolism. *ALDH2* rs671 p.Glu504Lys pathogenic mutant reduces the activity of ALDH2, which is associated with reduced risk of gout [41].In addition, the rs671 GA + AA genotype was found to be associated with a lower risk of gout, while alcohol and BMI abnormalities were associated with a higher risk of gout in Taiwan population. Moreover, BMI and alcohol have a significant interaction on the risk of gout in patients with GG and GA + AA [111].

### UA regulatory genes related to glycolysis

In humans, the disorder of glycometabolism can also indirectly affect purine metabolism, thus affecting SUA levels. For example, fructose can indirectly elevate SUA levels via increasing ATP degradation in the liver [112]. Moreover, many genes involved in glycometabolism (such as *GCKR*; *PKLR*; *MLXIPL*; *PRKAG2*; *NFAT5*; *NF4G*, etc.) indirectly affect SUA levels. Current studies have shown that among many genes involved in glycolysis, *GCKR* gene (located on chromosome 2p23.3) encoding glucokinase regulatory protein seems to be the most important gene affecting SUA levels. Its expression is regulated by lncRNAs ENST00000588707.1 and TCONS_00004187 [113]. Furthermore, *GCKR* gene mutations accelerate the transition from asymptomatic HUA to gout, and its SNP rs1260326 is associated with a higher risk of gout [114, 115]. Interestingly, *GCKR* interacts with alcohol to reduce the risk of gout [116].

## Conclusion

This paper reviews the susceptibility genes and their variants involved in UA transport on HUA and gout. We found that *SLC22A* family, *ABC* family and *SLC2A* family are the most studied gene families among many susceptible genes at present. Interestingly, *SLC22A* family gene mutations can not only increase the risk of HUA and gout, but also reduce SUA levels and even cause severe hypouricemia. Moreover, some SNPs of *SLC22A* family (such as rs121907896 and rs121907892) also have significant anti-gout effects.

In summary, genomic studies on UA metabolism contribute to an in-depth understanding of the pathogenesis of HUA and gout. Generally speaking, gene level changes often precede protein level in the process of disease occurrence and development. Therefore, the study of HUA and gout at the gene level is still an important direction of our future research. If we can identify the highly specific and sensitive gene markers of elevated SUA levels, then, it will provide great help for the early diagnosis of HUA and the prevention and targeted treatment of gout patients.

## Data Availability

Not applicable.

## Abbreviations

SUA: Serum uric acid
UA: Uric acid
HUA: Hyperuricemia
PRS1: Phosphoribosyl pyrophosphate synthase1
HPRT: Hypoxanthine–guanine phosophoribosyltransferase
PRPP: 5'-Phosphoribosyl-1'-pyrophosphate
IMP: Inosine monophosphate
GMP: Guanosine monophosphate
XO: Xanthine oxidase
LND: Lesch-Nyhan syndrome
APP: β-Amyloid precursor protein
ALDH16A1: Acetaldehyde dehydrogenase 16 family A1
OAT1/3/4/10: Organic anion transporter 1/3/4/10
SNP: Single nucleotide polymorphism
GWAS: Genome-wide association studies
RUE gout: Renal underexcretion gout
URAT1: Urate transporter 1
RHUC1: Hereditary renal hypouricemia type 1
GLUT9/12: Glucose transporter 9/12
RHUC2: Hereditary renal hypouricemia type 2
E4BP4: E4 promoter-binding protein 4
AKI: Acute renal injury
BCRP: Breast cancer resistance protein
MRP4: Multidrug resistance protein 4
NPT1/4/5: Sodium dependent phosphate transporter 1/4/5
PDZK1: PDZ domain-containing 1
HEK293 cells: Human embryonic kidney 293 cells
lncRNA: Long noncoding RNA
MCT9: Monocarboxylic acid transporter 9
ROL gout: Renal overload gout
CARMIL: Myosin I connexin
UMOD: Uromodulin
THP: Tamm-Horsfall protein
ADTKD-UMOD: Autosomal dominant tubulointerstitial kidney disease
FJHN: Familial juvenile hyperuricemia nephropathy
